# Quality-of-life assessment instruments for patients with vestibular schwannoma: A systematic review

**DOI:** 10.1016/j.bjorl.2025.101585

**Published:** 2025-03-21

**Authors:** Katarzyna Bieńkowska, Barbara Kostecka, Andrzej Kokoszka

**Affiliations:** Medical University of Warsaw, Department of Psychiatry, Warsaw, Poland

**Keywords:** Quality of life, Health-related quality of life, Vestibular schwannoma, Acoustic neuroma

## Abstract

**Objective:**

The European Association of Neuro-Oncology (EANO) indicated that different study designs and methodologies led to inconsistent conclusions about treatment methods and QoL in patients with Vestibular Schwannoma (VS). This study systematically reviews recent findings about QoL assessment instruments for patients with VS and provides recommendations for the measurement of QoL with standardized psychometric tools.

**Methods:**

This study was conducted based on the PRISMA 2020 guideline for reporting systematic reviews. The key words used were as follows: quality of life, health-related quality of life, vestibular schwannomas, acoustic neuroma. Studies were included when the following eligibility criteria were met.

**Results:**

Our search finally yielded 48 articles published in years 2014–2024 that measured QoL in patients with VS using standardized psychometric tools. The studies examined various dimensions of QoL. The most frequently used disease-specific questionnaire was the Penn Acoustic Neuroma Quality of Life (PANQOL). As a generic tool, authors preferred to use the Short-Form 36-Item Health Survey (SF-36).

**Conclusion:**

Recent findings and data on Quality of Life (QoL) tools for patients with VS emphasize the importance of using standardized and disease specific tool like PANQOL, due to their frequent use and large amount of patient data. Although the SF-36 is commonly used for general assessments, it may not be sensitive enough for a complete QoL evaluation and should not be the only tool used. The GBI is effective for assessing changes after specific medical treatments. Using methods similar to those in past studies helps medical professionals make reliable conclusions about QoL and evaluate the effects of different treatments in VS patients.

## Highlights

• Simultaneously use disease specific and generic QoL tools in VS patients.
• Use the SF-36 as a generic tool and PANQOL as a disease-specific tool in VS studies.
• Including medical data, questionnaires scores referring to VS to assess HRQOL.

## Introduction

Currently, the measure of success in medicine is not only treatment but also the improvement of Health-Related Quality of Life (HRQoL) in psychological and social aspects.^1^ Over the last decade, medical community showed growing interest in developing methods for measuring and comparing objective patients’ outcomes, coupled with subjective patients’ assessment.^2^ Among a variety of tools, questionnaires enable health-care professionals to quickly get to know the patient’s perspective on experienced symptoms. Two types of standardized questionnaires can be used for QoL assessment: generic or disease specific. A generic questionnaire may be less sensitive in the assessment of changes related to disease or treatment. Therefore, disease-specific questionnaires are dedicated for patients diagnosed with a particular entity, as they are designed with focus on symptoms or a health aspect that may be affected by the disease such as Vestibular Schwannoma (VS).

### Objective

The European Association of Neuro-Oncology (EANO) indicated that different study designs and methodologies led to inconsistent conclusions about treatment methods and QoL in patients with VS.^3^ This study systematically reviews recent findings about QoL assessment instruments for patients with VS and provides recommendations for the measurement of QoL with standardized psychometric tools.

## Methods

### Study quality

This study was conducted based on the PRISMA 2020 guideline for reporting systematic reviews.^4^ The methodological quality of the studies was assessed with an applicable CASP Systematic Review Checklist (Critical Appraisal Skills Programme) to assess risk of bias.

### Search strategy and selection process

**Fig. 1:**
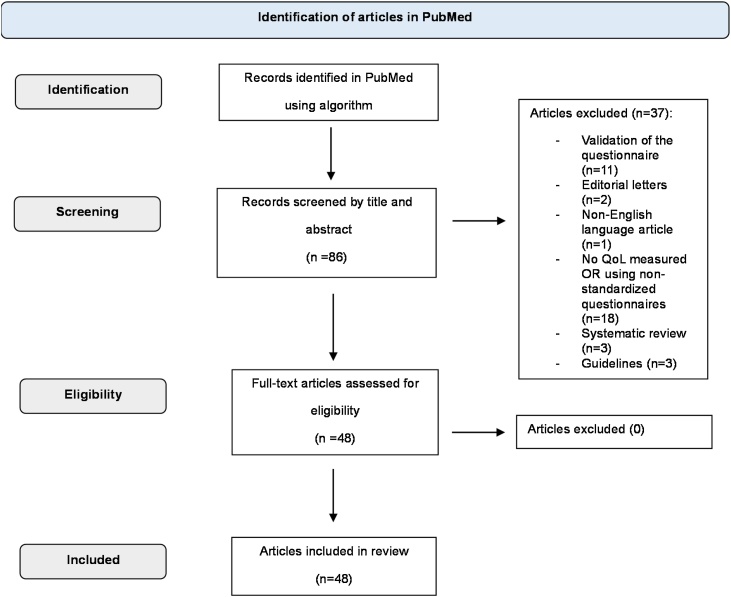
PRISMA flow diagram of the search strategy.

A systematic literature search was conducted from January, 4 to February, 25, 2024, to identify articles about QoL assessment instruments for patients with VS published between 2014 and 2024 in the PubMed database. The following search terms were used: “quality of life” OR “health-related quality of life” AND “vestibular schwannomas” OR “acoustic neuroma”. The key words used were as follows: quality of life, health-related quality of life, vestibular schwannomas, acoustic neuroma. Titles and abstracts were independently screened by two reviewers (KB and BK). Then, full-text screening was conducted by one reviewer (KB) and discussed with the second reviewer (BK). When full-text inclusion was debatable, the third reviewer made the final decision about inclusion (AK). For details, see Fig. 1.

### Study selection criteria

Studies were included when the following eligibility criteria were met studies published in English, assessment of QoL conducted in adult patients with VS using standardized validated questionnaires (generic and disease-specific tools). The exclusion criteria were as follows: studies published in languages other than English, unpublished manuscripts and conference materials, case reports, studies using unvalidated instruments.

## Results

### Characteristics of the studies

Our search finally yielded 48 articles published in years 2014–2024 (Table 1) that measured QoL in patients with VS using standardized psychometric tools. The studies examined various dimensions of QoL. We identified 12 tools (5 disease-specific questionnaires and 7 generic tools) used in studies involving patients with VS and the tendency has been observed in studies to use both disease-specific and generic tools to evaluate QoL.

**Table 1:** The list of studies measuring quality of life in patients with vestibular schwannomas.

Study	Questionnaire	Group (n)
Lazak et al., 2024^5^	Penn Acoustic Neuroma Quality of Life (PANQOL)	43
	World Health Organization Quality of Life-BREF (WHOQOL-BREF)	
Quimby et al., 2023^6^	Penn Acoustic Neuroma Quality of Life (PANQOL)	296
Nowacka et al., 2023^7^	Penn Acoustic Neuroma Quality of Life (PANQOL)	52
Marinelli et al., 2023^8^	Penn Acoustic Neuroma Quality of Life Scale the Mayo Clinic Vestibular Schwannoma Quality of Life Index	244
Dhayalan et al., 2023^9^	Penn Acoustic Neuroma Quality of Life (PANQOL)	100
Neve et al., 2023^10^	Penn Acoustic Neuroma Quality of Life (PANQOL)	536
Lucidi et al., 2022^11^	Penn Acoustic Neuroma Quality of Life (PANQOL)	111
	Glasgow Benefit Inventory (GBI)	
Küchler et al., 2022^12^	Heidelberg SYQOL Inventory (SYQOL)	168
Foscolo et al., 2022^13^	Penn Acoustic Neuroma Quality of Life (PANQOL)	31
Fuentealba-Bassaletti et al., 2022^14^	Penn Acoustic Neuroma Quality of Life (PANQOL)	287
Bender et al., 2022^15^	Short-Form 36-item (SF-36)	43
	Glasgow Benefit Inventory (GBI)	
North et al., 2022^16^	Penn Acoustic Neuroma Quality of Life (PANQOL)	326
Sergi et al., 2022^17^	Short-Form 12-item (SF-12)	132
Windisch et al., 2021^18^	Short-Form 12-item (SF-12)	175
Chweya et al., 2021^19^	Penn Acoustic Neuroma Quality of Life (PANQOL)	1362
Pattankar et al., 2021^20^	Short-Form 36-item (SF-36)	64
	Penn Acoustic Neuroma Quality of Life (PANQOL)	
Said et al., 2021^21^	Penn Acoustic Neuroma Quality of Life (PANQOL)	138
Carlson et al., 2021^22^	Penn Acoustic Neuroma Quality of Life (PANQOL)	244
Pruijn et al., 2021^23^	Short-Form 36-item (SF-36)	174
	Penn Acoustic Neuroma Quality of Life (PANQOL)	
Brown et al., 2020^24^	Penn Acoustic Neuroma Quality of Life (PANQOL)	43
Miller et al., 2020^25^	Penn Acoustic Neuroma Quality of Life (PANQOL)	134
Hebb et al., 2019^26^	Hearing Handicap Inventory	210
Kojima et al., 2019^27^	Short-Form 36-item (SF-36)	72
Miller et al., 2019^28^	Penn Acoustic Neuroma Quality of Life (PANQOL)	123
Klersy et al., 2018^29^	Short-Form 36-item (SF-36)	65
Deberge et al., 2018^30^	Short-Form 36-item (SF-36)	142
Lodder et al., 2018^31^	Penn Acoustic Neuroma Quality of Life (PANQOL)	359
Carlson et al., 2018^32^	Penn Acoustic Neuroma Quality of Life (PANQOL)	1288
Glaas et al., 2018^33^	Penn Acoustic Neuroma Quality of Life (PANQOL)	72
Link et al., 2018^34^	Short-Form 36-item (SF-36)	143
	Penn Acoustic Neuroma Quality of Life (PANQOL)	
	Patient-Reported Outcomes Measurement Information System (PROMIS-10)	
Berkowitz et al., 2017^35^	Short-Form 36-item (SF-36)	353
Soulier et al., 2017^36^	Penn Acoustic Neuroma Quality of Life (PANQOL)	1208
Tveiten et al., 2017^37^	Hearing Handicap Inventory	539
	Short-Form 36-item (SF-36)	
	Penn Acoustic Neuroma Quality of Life (PANQOL)	
Foley et al., 2017^38^	Functional Assessment of Chronic Illness Therapy-Brain Questionnaire	83
Broomfield et al., 2017^39^	Short-Form 36-item (SF-36)	500
Ribeyre et al., 2016^40^	World Health Organization Quality of Life-BREF (WHOQOL-BREF)	26
Čada et al., 2016^41^	Glasgow Benefit Inventory (GBI)	10
	Glasgow Health Status Inventory (GHSI)	
Kim et al., 2015^42^	Short-Form 36-item (SF-36)	108
Turel et al.,2015^43^	Short-Form 36-item (SF-36)	100
	Glasgow Benefit Inventory (GBI)	
McLaughlin et al., 2015^44^	Penn Acoustic Neuroma Quality of Life (PANQOL)	186
Carlson et al., 2015^45^	Short-Form 36-item (SF-36)	538
	Penn Acoustic Neuroma Quality of Life (PANQOL)	
Carlson et al., 2015^46^	Short-Form 36-item (SF-36)	538
	Penn Acoustic Neuroma Quality of Life (PANQOL)	
Carlson et al., 2015^2^	Short-Form 36-item (SF-36)	642
	Penn Acoustic Neuroma Quality of Life (PANQOL)	
	Glasgow Benefit Inventory (GBI)	
	Patient-Reported Outcomes Measurement Information System (PROMIS-10)	
Jufas et al., 2015^47^	Short-Form 36-item (SF-36)	223
Wangerid et al., 2014^48^	EuroQoL-5 Dimension (EQ-5D)	128
Scheich et al., 2014^49^	Short Form-36 (SF-36)	91
Robinett et al., 2014^50^	Penn Acoustic Neuroma Quality of Life (PANQOL)	294
van Leeuwen et al., 2014^51^	Penn Acoustic Neuroma Quality of Life (PANQOL)	254

The most frequently used disease-specific questionnaire was the Penn Acoustic Neuroma Quality of Life (PANQOL) and has been used in 29 studies, with results from 9831 patients. The extensive application of PANQOL across numerous studies and its assessment of a large patient cohort make it a significant instrument for evaluating quality of life in patients with VS. As a generic tool, authors preferred to use the Short-Form 36-Item Health Survey (SF-36). SF-36 utilized in 16 studies, with results from 3797 patients. The SF-36 is a widely recognized tool for assessing general health status. Its frequent use and substantial patient sample size highlight its relevance and reliability in measuring health outcomes in this patient group. In 5 studies with results from 906 patients the Glasgow Benefit Inventory (GBI) was used for the post-intervention measurement of change related to a specific medical intervention, especially an otolaryngological intervention.

Moreover, in a few studies, the following tools were used: World Health Organization Quality of Life-BREF (WHOQOL-BREF) (2), 10-item Patient-Reported Outcomes Measurement Information System (PROMIS-10) (2), Hearing Handicap Inventory (2) Short Form-12 (SF-12) (2), Mayo Clinic Vestibular Schwannoma Quality of Life Index (VSQOL) (1) SYQOL (1) EuroQoL-5 Dimension (EQ-5D) (1), Functional Assessment of Chronic Illness Therapy-Brain Questionnaire (FACIT) (1), and Glasgow Health Status Inventory (GHSI) (1). For details, see Table 2.

**Table 2:** Quality-of-life assessment instruments used in studies on the population of patients treated for vestibular schwannomas.

Quality of life assessment instruments used in for patients with vestibular schwannomas	Number of studies applying the tool	Total number of patients
Penn Acoustic Neuroma Quality of Life (PANQOL)^2^, ^5^, ^6^, ^7^, ^9^, ^10^, ^11^, ^13^, ^14^, ^16^, ^19^, ^20^, ^21^, ^22^, ^23^, ^24^, ^25^, ^28^, ^31^, ^32^, ^33^, ^34^, ^36^, ^37^, ^44^, ^45^, ^46^, ^50^, ^51^	29	9831
Short-Form 36-item (SF-36)^2^, ^15^, ^20^, ^23^, ^27^, ^29^, ^30^, ^34^, ^35^, ^37^, ^39^, ^42^, ^43^, ^45^, ^46^, ^47^, ^49^	16	3797
Glasgow Benefit Inventory (GBI)^2^, ^11^, ^15^, ^41^, ^43^	5	906
World Health Organization Quality of Life-BREF (WHOQOL-BREF)^5^, ^40^	2	785
Patient-Reported Outcomes Measurement Information System (PROMIS-10)^2^, ^34^	2	749
Hearing Handicap Inventory 26, 37	2	307
Short Form-12 (SF-12)^17^, ^18^	2	244
Vestibular Schwannoma Quality of Life Index^8^	1	168
SYQOL^12^	1	128
EuroQoL-5 Dimension (EQ-5D)^48^	1	83
Functional Assessment of Chronic Illness Therapy-Brain Questionnaire (FACIT)^38^	1	69
Glasgow Health Status Inventory (GHSI)^41^	1	10

We identified 12 tools used in studies involving patients with VS however, considering the frequency of usage and the sample sizes from these studies and number of studies, we decided to focus on and compare the 3 most commonly used tools, which have been validated on the largest number of participants and studies: PANQOL, SF-36 and GBI.

### Characteristics of the tools used in studies

Findings from the reviewed studies showed that two types of standardized questionnaires have been used in patients with VS for QoL assessment: generic or disease specific. We identified 5 disease-specific questionnaires and 6 generic tools mainly used in the studies.

### Disease-specific questionnaires

PANQOL is a questionnaire used to evaluate QoL in patients with VS. The tool consists of 26 items with responses ranging from 1 (strongly disagree) to 5 (strongly agree). Domain scores are obtained by averaging the responses for items assigned to individual domains: hearing, balance, facial function, pain, anxiety, energy and general health. The values of test-retest reliability and internal consistency were high.^2^

Vestibular Schwannoma Quality of Life Index is a tool consisting of 40 items that evaluates the impact of VS diagnosis and management on the patient’s QoL, treatment satisfaction, and employment. Domain scores range from 0 (worst) to 100 (best).^52^

SYQOL, a questionnaire called Heidelberg SYQOL Inventory, is applied in a large cohort of patients treated with stereotactic radiosurgery and includes questions on symptom control, outcome, and QoL for self-reported outcome. This tool refers to typical symptoms, side effects, long-term outcome, and QoL of patients with VS prior to radiotherapy and after treatment.^53^ No adaptation or validation process for this questionnaire has been found.

HHI is a questionnaire used to evaluate the effects of hearing loss on two subscales: emotional consequences of hearing loss and its social and situational effects. All of the 25-items are assessed with an ordered response with three possible answers: “yes”, “sometimes”, or “no”. This questionnaire has not been adequately used for QoL measurement in studies.^54^

FACIT is a 50-item questionnaire used to evaluate HRQoL across various domains such as physical well-being, social/family well-being, emotional well-being, functional well-being, and malignant brain tumor subscales. There are five response options for each item, with scores ranging from 0 to 4-points.^55^, ^56^

### Generic QoL questionnaires

SF-36 is a very popular questionnaire for evaluation of HRQoL, which consists of 35 items. The tool measures 8 scales: Physical Functioning (PF), Role-Physical (RP), Bodily Pain (BP), General Health (GH), vitality (VT), Social Functioning (SF), Role-Emotional (RE), and Mental Health (MH). There are two distinct concepts measured by the SF-36: a physical (Physical Component Summary [PCS]) and mental dimension (Mental Component Summary [MCS]).^57^ The SF-12 covers the same domains as SF-36 with substantially fewer questions.

SF-12 covers the same domains as SF-36 with substantially fewer questions.

WHOQOL-BREF is a 26-item questionnaire with responses ranging from 1 to 5, which consists of 4 domains: physical health, psychological health, social relationships, and environmental health; it also contains QoL and general health items.^58^

PROMIS-10 is a questionnaire used to measure health status across multiple domains (physical, mental, and social) of HRQoL from the patient’s perspective in a variety of chronic diseases. Responses range from 1 (never) to 5 (always).^59^

EQ-5D is an instrument that evaluates the generic QoL. The descriptive system comprises five dimensions: mobility, self-care, usual activities, pain/discomfort, and anxiety/depression. Each dimension has 5 levels from no problems until extreme problems.^60^

GHSI is an 18-item questionnaire based on a 5-point Likert scale from much worse to much better, used to assess health status by measuring the effect of a health problem on QoL. Questions are grouped in 3 subscales: general, social, and physical health.^61^, ^62^

GBI is designed for use only once after an intervention to evaluate the change related to specific medical interventions, especially those otolaryngological. GBI consists of 18 items based on a 5-point Likert scale from much worse to much better.^63^

## Discussion

Findings from the reviewed studies consistently indicate discrepancies in methods used to evaluate QoL in patients with VS, which is consistent with data reported by the EANO.^3^ Undoubtedly, an appropriate instrument adaptation should comply with the following generally accepted criteria: standardization, reliability, and validity. Therefore, in this literature review, we included only studies that measured QoL with standardized validated questionnaires (generic and disease-specific tools). We identified 12 tools used in studies involving patients with VS. The tools used in the studies were characterized by good or strong psychometric properties. However, taking into account the frequency with which these tools were used, and the sample sizes involved in the studies, we decided to concentrate on and compare the three most frequently utilized tools. These tools have been validated with the largest number of participants, making them particularly significant for our analysis.

The PANQOL was the most frequently used disease-specific questionnaire in 29 studies including 9831 results from patients. The PANQOL was the most frequently used disease-specific questionnaire in 29 studies including 9831 results from patients. If we focus on the evaluation of Quality of Life (QoL) related to VS symptoms, it is reasonable to assess the patient’s perspective using a disease-specific questionnaire like the PANQOL. This tool is specifically designed to capture the unique challenges and issues faced by patients with VS, providing a comprehensive understanding of how the condition impacts their daily lives. By tailoring the assessment to the specific symptoms and experiences associated with VS, PANQOL allows for a more accurate and meaningful evaluation of the patient's quality of life. This approach ensures that the nuances of living with VS are effectively captured, facilitating targeted interventions and personalized care plans. Also, the PANQOL has been adapted to different languages and cultures and widely used in the medical environment. The results of various cross-cultural adaptations showed that the PANQOL is a reliable and valid questionnaire^64^, ^65^, ^66^, ^67^, ^68^, ^69^ and meets generally accepted psychometric criteria. By utilizing the PANQOL questionnaire, the authors of the 29 previously published studies were able to compare Quality of Life (QoL) outcomes and evaluate the impact of various Vestibular Schwannoma (VS) treatment options from the patient’s perspective. The latest disease-specific questionnaire, also developed by Carlson et al. in, is the Vestibular Schwannoma Quality of Life Index.^52^ Both tools exhibit strong psychometric properties; however, PANQOL has been adapted into more languages and has collected more patient data and has been utilized in research involving various treatment options for VS. The EANO emphasized the importance of using similar study designs and methodologies to achieve consistent conclusions regarding treatment methods and quality of life in patients with VS.^3^ Utilizing a standardized questionnaire allowed researchers to gather and compare data more effectively, resulting in valuable insights and conclusions.

As a generic tool for the assessment of QoL, authors preferred to use the SF-36 (16 studies with results from 3797 patients), which also demonstrates good psychometric properties^70^ and is very popular in different medical environments. The SF-36 is available in many language versions, which is a strong argument for its use. It has been applied to large numbers of patients and is designed to assess HRQOL, taking into account various health-related subscales. However, when used alone, the SF-36 may not provide reliable results specifically related to the QoL issues unique to VS. It may be challenging to determine whether a lower quality of life is due to VS-specific difficulties or issues in other areas. Bess reached similar conclusions, noting that a review of studies on QoL measures revealed that existing generic functional health status tools lack the sensitivity needed to detect clinically significant improvements in patients with hearing impairment.^71^ Rostkowska et al., mentioned in their literature review that the SF-36 questionnaire is not well adjusted for patients with hearing loss (especially after cochlear implantation), and generic tools are not sensitive enough for the assessment of QoL and therefore should not be used as the only tool for QoL evaluation.^72^ This is consistent with findings from studies conducted in the population of patients with VS. However, according to Marra et al., general measures allow for comparisons across different disease conditions, which can offer valuable data for making decisions related to health policy and resource distribution.^73^

The third most frequently used questionnaire is the GBI, which differs in its measurement focus compared to PANQOL. The GBI is specifically designed to assess changes following a medical intervention, particularly in the field of otolaryngology. Using this tool, the patient can evaluate how the direct intervention has contributed to either the improvement or deterioration of their condition. It has been popularized since its design and used as a generic patient-recorded outcome measure in over 100 surgical studies for otorhinolaryngological conditions.^74^ The GBI is intended for single use after intervention as a measure of HRQoL and patient’s benefit. The original version of the GBI is a reliable, sensitive, and validated post-intervention questionnaire. Moreover, confirmatory and exploratory factor analyses were conducted in a group of 1980 adult patients who had complete GBI data and who underwent an active (medical or surgical) intervention.^75^ Also, the GBI has been adapted in and translated into numerous languages.

**Fig. 2:**
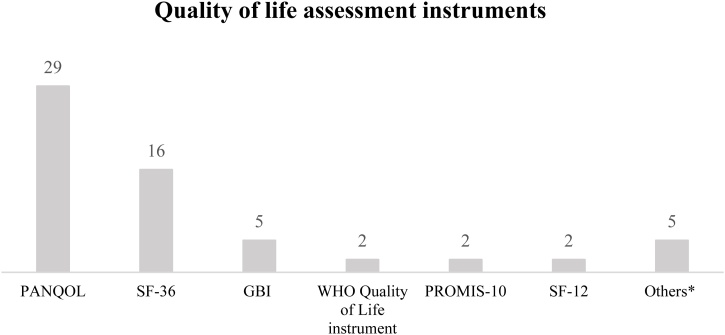
Quality-of-life assessment instruments used in studies on the population of patients treated for vestibular schwannoma.

**Fig. 3:**
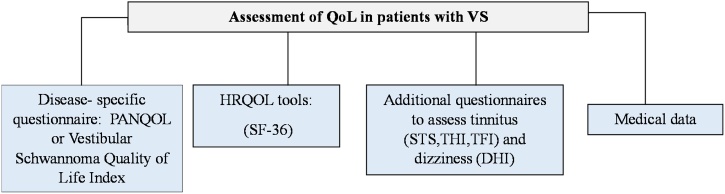
Recommendations for the assessment of quality of life in patients with vestibular schwannoma.

Findings from the studies showed that there was a tendency to simultaneously use both types of questionnaires to evaluate QoL: disease-specific and generic tools. Therefore, to adequately evaluate QoL, we would recommend using both questionnaires (Fig. 2). The generic questionnaire may be less sensitive in the assessment of changes related to disease or treatment. Moreover, OoL is a subjective construct, and many factors may have an impact on it in the patient’s life (see Fig. 3). Using HRQoL questionnaires, it is possible to assess the impact of disease and treatment on the patient’s daily life not only in terms of physical health but also in psychological and social areas. Moreover, using both types of questionnaires, we can indicate what is an effect of VS diagnosis and presence of symptoms, and what is an effect of difficulties in other areas, such as family life and financial situation, work environment, comorbidities. Additionally, we would recommend including medical data and results from supplementary questionnaires referring to VS symptoms, such as tinnitus, vertigo, and dizziness, to assess patients’ HRQoL.

### Limitations

Admittedly, our systematic review has some limitations. We performed a comprehensive search using only a single multidisciplinary data source. Additionally, we used hand searching to analyze the reference lists of the relevant articles (2691 studies identified). Our conclusions were based on data from studies identified from one database and only articles published in English were included; of note, we identified one relevant study written in another language (German).

## Conclusions

Recent findings and data on Quality of Life (QoL) tools for patients with VS emphasize the importance of using standardized and disease specific tool like PANQOL, due to their frequent use and large amount of patient data. Although the SF-36 is commonly used for general assessments, it may not be sensitive enough for a complete QoL evaluation and should not be the only tool used. The GBI is effective for assessing changes after specific medical treatments. Using methods similar to those in past studies helps medical professionals make reliable conclusions about QoL and evaluate the effects of different treatments in VS patients.

## Funding

This research did not receive any specific grant from funding agencies in the public, commercial, or not-for-profit sectors.

## Declaration of competing interest

The authors declare no conflicts of interest.
